# *PIN3* positively regulates the late initiation of ovule primordia in *Arabidopsis thaliana*

**DOI:** 10.1371/journal.pgen.1010077

**Published:** 2022-03-04

**Authors:** Li-Qin Hu, Jin-Hui Chang, Shi-Xia Yu, Yu-Tong Jiang, Rong-Han Li, Ji-Xuan Zheng, Yan-Jie Zhang, Hong-Wei Xue, Wen-Hui Lin

**Affiliations:** 1 School of Life Sciences and Biotechnology, The Joint International Research Laboratory of Metabolic and Developmental Sciences, Shanghai Collaborative Innovation Center of Agri-Seeds/Joint Center for Single Cell Biology, Shanghai Jiao Tong University, Shanghai, China; 2 Zhiyuan College, Shanghai Jiao Tong University, Shanghai, China; 3 Shanghai Collaborative Innovation Center of Agri-Seeds/Joint Center for Single Cell Biology, School of Agriculture and Biology, Shanghai Jiao Tong University, Shanghai, China; Peking University, CHINA

## Abstract

Ovule initiation determines the maximum ovule number and has great impact on seed number and yield. However, the regulation of ovule initiation remains largely elusive. We previously reported that most of the ovule primordia initiate asynchronously at floral stage 9 and PINFORMED1 (PIN1) polarization and auxin distribution contributed to this process. Here, we further demonstrate that a small amount of ovule primordia initiate at floral stage 10 when the existing ovules initiated at floral stage 9 start to differentiate. Genetic analysis revealed that the absence of *PIN3* function leads to the reduction in pistil size and the lack of late-initiated ovules, suggesting *PIN3* promotes the late ovule initiation process and pistil growth. Physiological analysis illustrated that, unlike picloram, exogenous application of NAA can’t restore these defective phenotypes, implying that PIN3-mediated polar auxin transport is required for the late ovule initiation and pistil length. qRT-PCR results indicated that the expression of *SEEDSTICK* (*STK*) is up-regulated under auxin analogues treatment while is down-regulated in *pin3* mutants. Meanwhile, overexpressing *STK* rescues *pin3* phenotypes, suggesting *STK* participates in PIN3-mediated late ovule initiation possibly by promoting pistil growth. Furthermore, brassinosteroid influences the late ovule initiation through positively regulating *PIN3* expression. Collectively, this study demonstrates that *PIN3* promotes the late ovule initiation and contributes to the extra ovule number. Our results give important clues for increasing seed number and yield of cruciferous and leguminous crops.

## Author summary

Ovule is the precursor of seed. Ovule initiation determines maximum ovule number and has great impact on seed number and yield. Understanding the process and regulation of ovule initiation has both scientific significance and potential application. Although the process and its related genes have been reported, the underling mechanisms remains elusive. We previously reported the asynchronous ovule initiation and the hormones integration in regulating ovule initiation. In this study, we found there is late ovule initiation at floral stage 10 in the wild-type Arabidopsis when the existing ovules initiated at floral stage 9 start to differentiate, which contributes to around 10% extra ovule number. We identified *pin3* mutants lacking late-initiated ovules, indicating *PIN3* facilitates the late ovule initiation. Exogenous application of auxin analogues revealed that polar auxin transport mediated by PIN3 is essential for the late ovule initiation. *STK*, the positive regulator of ovule initiation, involves in PIN3-mediated late ovule initiation. BR influences the late ovule initiation through positively regulating *PIN3* expression. Taken together, we describe the intact process of ovule initiation and demonstrate the late ovule initiation contributes to the extra ovule number, which provides promising strategies for yield improvement of cruciferous and leguminous crops.

## Introduction

There are many seeds in one fruit in Arabidopsis and some important crops, such as leguminous and cruciferous crops. The ovule is the precursor of the seed, and its initiation process determines the maximum ovule number per flower and has great impact on the maximum seeds per fruit, making it an important factor affecting the final seed yield of these plants.

In *A*. *thaliana*, the ovule primordia arise asymmetrically at floral stages 8–9 by the periclinal cell divisions within the subepidermal tissue of the placenta, which develops from the two opposing meristematic ridges (also called carpel margin meristems, CMMs) and differentiates along the length of the septum adjacent to the pistil walls at floral stage 8 (floral developmental stages according to [1]) [2–13]. The ovule primordia protrusion can be considered as a type of lateral organ initiation [5,14]. To date, several key genes and genetic regulatory networks related to ovule primordia identity and initiation have been characterized. The MADS box genes *APETALA2* (*AP2*), *AGAMOUS* (*AG*), *SHATTERPROOF1/2* (*SHP1/2*), *SEPALLATA1/2/3* (*SEP1/2/3*) and *SEEDSTICK* (*STK*) encode crucial regulators of ovule identity and initiation through affecting carpel development [15–19]. Among these genes, *STK* specifically expresses in placentae and ovules, and is regulated by the GA-binding protein BPC1 [18,20,21]. Additionally, mutations in other pivotal regulators, including *HUELLENLOS* (*HLL)*, *BELL1* (*BEL1*), and *AINTEGUMENTA* (*ANT*), decrease the ovule number [22–26]. *CUC1* and *CUC2* function redundantly controlling septal fusions and ovule boundaries (the interval between ovules), thereby influencing ovule identity. Moreover, *cuc1 cuc2* double mutant produce fewer ovule primordia than wild-type [27,28].

Plant hormones have important regulatory effects on the ovule number. A double mutant (*yucca1 yucca4*) in which auxin synthesis is adversely affected produces fewer ovules because of a compromised local auxin response [29]. A similar phenotype was also observed in the *mp S319* mutant with a weakly mutated *MONOPTEROS* (*MP*), encoding a transcription factor of the auxin response factor (ARF) family [30–32]. Polar auxin transport is essential for the regulation of ovule initiation since the partial loss-of-function *pin1-5* mutant showed dramatically reduced ovule number [11,28,33]. Moreover, MP directly targets *CUC1* and *CUC2* to control *PIN1* expression and localization during the formation of ovule primordia [28]. Currently, we reported that the auxin flow mediated by the dynamic polar localization of auxin efflux carrier PIN1, leading to the formation of auxin maxima, is essential for asynchronous ovule initiation [11].

Cytokinin (CK) has also been demonstrated to regulate the ovule primordia formation. In *cytokinin oxidase/dehydrogenase3* (*ckx3) ckx5* double mutant, the ovule number was increased due to the raised CK content [34]. By contrast, the number of ovule primordia decreases substantially in the *cytokinin response1-12*(*cre1-12*) *histidine kinase2-2* (*ahk2-2*) *ahk3-3* and *Arabidopsis thaliana response regulator1* (*arr1*) *arr10 arr12* triple mutants, which exhibit decreased responsiveness to CK because of the altered auxin fluxes resulting from the modulated *PIN1* expression during ovule development [33,35–37].

Brassinosteroid (BR) positively regulates the ovule number as well. Earlier research demonstrated that BR-deficient mutants, including *cpd*, *bri1-116*, *bri1-5*, *bin2-1* and *det2-1*, have shortened pistils and produce relatively few ovules [38,39], whereas the enhanced BR signaling in the *bzr1-1D* mutant lengthens the pistil and significantly increases the ovule number by up-regulating the expression of *HLL* and *ANT* [39]. The shortened pistils of the *bin2-1* mutant are partial result of the decreased auxin signaling [40], which is consistent with our report that increased BR could enhance auxin response during ovule initiation [11].

Considered together, the integration of multiple plant hormones regulates the ovule initiation. Furthermore, the auxin peaks directly mediate this process. Polar distribution of PIN proteins in the cell membrane enables the directional transport of intercellular auxin [41]. Among the eight members of the PIN family (*PIN1–PIN8*) in *A*. *thaliana* [42], *PIN1* and *PIN3* are mainly expressed in developing ovules [4,28,43,44]. Besides, *PIN1* is required for ovule patterning and female gametophyte development during the later ovule developmental stages [33], while PIN3 may not play predominant role since the *pin3-4* mutant shows normal ovule patterning and female gametogenesis (the publications of other alleles of *pin3* mutants have not mentioned the defective ovule development) [43,45]. However, whether *PIN3* contributes to the initiation of ovule primordia still remains unknown.

Our previous work has elucidated the two groups of asynchronous ovule primordia initiation at floral stage 9 [11]. Basically, the first group of ovule primordia protrude firstly. Along with the placenta elongation, the size of interval between existing ovules enlarges, and the second group of ovule primordia initiate from these boundary regions. These two group ovules grow to similar size and shape and begin differentiating at floral stage 10. In this study, we further illustrated that most of ovules (around 90%) initiated early (in two groups) and the remaining 10% ovules protruded late. Further investigations revealed that *pin3* mutants showed reduced pistil size and lacking late ovule initiation, leading to the specifically decreased extra ovule number. PIN3-mediated polar auxin transport is crucial for the late ovule initiation and *STK* involves in this process. In addition, BR participated in ovule initiation by positively regulating auxin signaling and *PIN3* expression. Taken together, our results describe the late process of ovule initiation in *A*. *thaliana*, demonstrate the regulatory mechanism of the late ovule initiation, and provide clues for increasing ovule number and seed yield.

## Results

### New ovule primordia form at floral stage 10 in the wild-type

**Fig 1:**
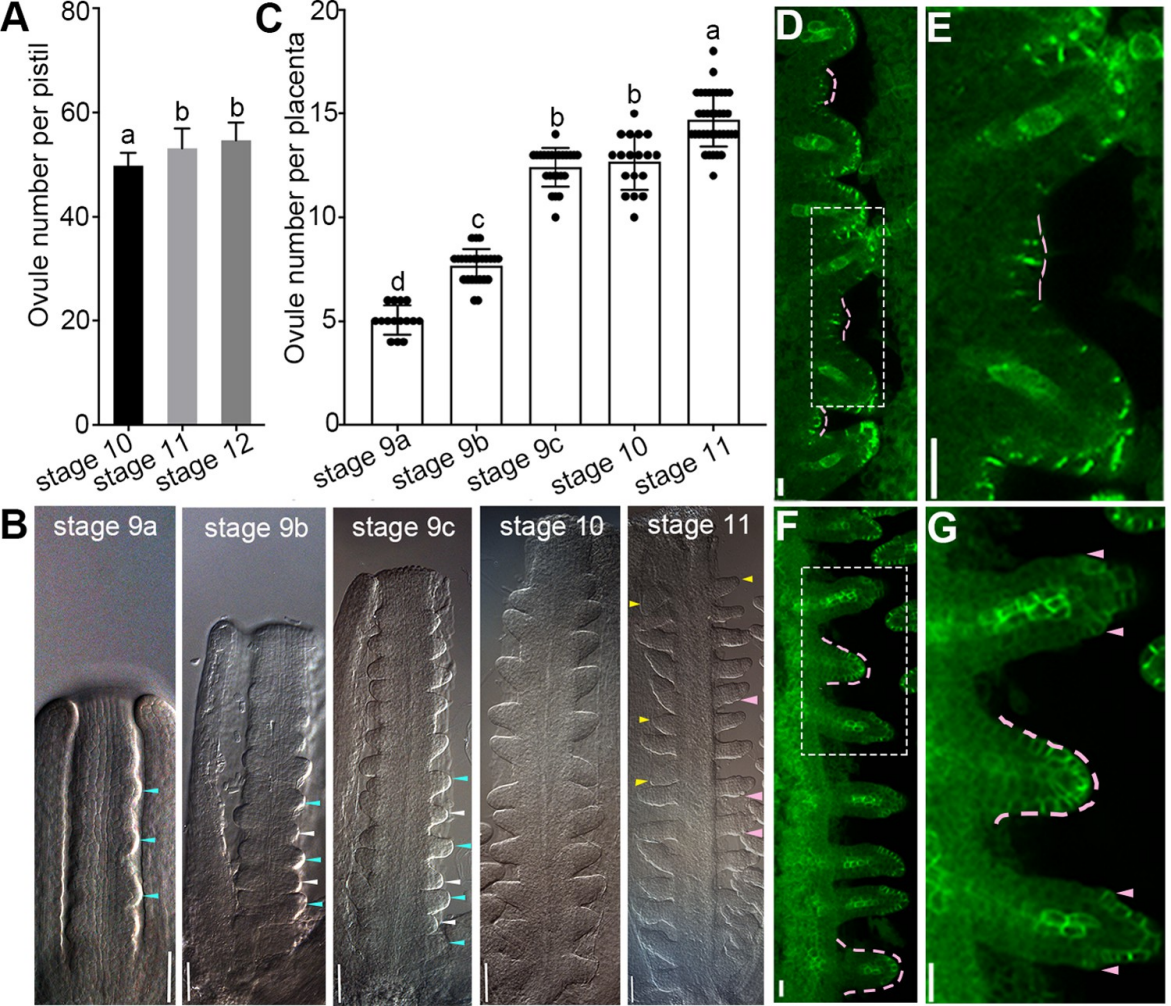
New ovule primordia initiate at floral stage 10 in the wild-type placentae. (A) Ovule number per pistil. (B) Differential interference contrast (DIC) images showing the asynchronous initiation of ovule primordia at floral stages 9a-11. (C) Ovule number per placenta. (D-G) Young ovule primordia are indicated by *pPIN1*::*PIN1-GFP* reporter at floral stage 10 (D-E) and stage 11 (F-G). (E) Magnified view indicated by dashed rectangle in (D) showing young ovule primordia at stage 1-I appearance between existing ovules at stage 2-I. (G) Magnified view indicated by dashed rectangle in (F) displaying young ovule at stage 2-I while adjacent ovules at stage 2-III. Blue and white arrows indicate the first and second group of ovule primordia, respectively (B, stages 9a, 9b, 9c). Yellow arrows (B, stage 11) and pink dashed lines (D-G) indicate the young ovules. Pink arrows indicate the integument primordia (B,G). Data are presented as the mean ± SD (n = 15). Lowercase letters indicate the significant differences revealed by one-way ANOVA (P < 0.05). Bars: 20 μm (B) and 10 μm (D-G).

Our previous study demonstrated that ovule primordia initiated asynchronously at floral stage 9 in the wild-type Arabidopsis [11]. Here, our further observations showed that the ovule number is significantly increased at floral stage 11 compared to stage 10. And the ovule number at floral stage 11 is equal to the total number of ovule primordia at floral stage 12 (Fig 1A). These results implied that there are new ovule primordia formation at floral stages 10–11. Therefore, we systematically analyzed the ovule primordia initiation process from floral stages 9a to 11 in the wild-type plants using differential interference contrast (DIC) microscopy (substages 9a, 9b, 9c according to [11]). The results indicated that the initiation of ovule primordia and its number at floral stages 9a–10 was consistent with previously published data (Fig 1B and 1C) [11]. Notably, a few finger-shaped ovules at ovule developmental stage 2-I (ovule developmental stages according to [2]) are observed at floral stage 11, while the older ovules at stage 2-III begin to develop integuments (Fig 1B), implying young ovule primordia start to protrude at floral stage 10. Consistent with prior reports [11,28,46,47], the expression of *pPIN1*::*PIN1-GFP* reporter [48] further revealed that some ovule primordia at stage 1-I appear at floral stage 10 (Fig 1D and 1E), and develop to the ovule primordia at stage 2-I at floral stage 11 (Fig 1F and 1G), which grow more slowly than the first two group ovules (Fig 1B, 1F and 1G). Compared with the regular ovule initiation of the first two groups, these young ovules initiated from only a few intervals and represented about 10% of the total number of ovule primordia. Therefore, we defined the two rounds of ovule initiation at floral stage 9 as the early ovule initiation (main process) and the later random initiation at floral stage 10 as the late ovule initiation (extra process).

### *PIN3* regulates ovule number and pistil growth

**Fig 2:**
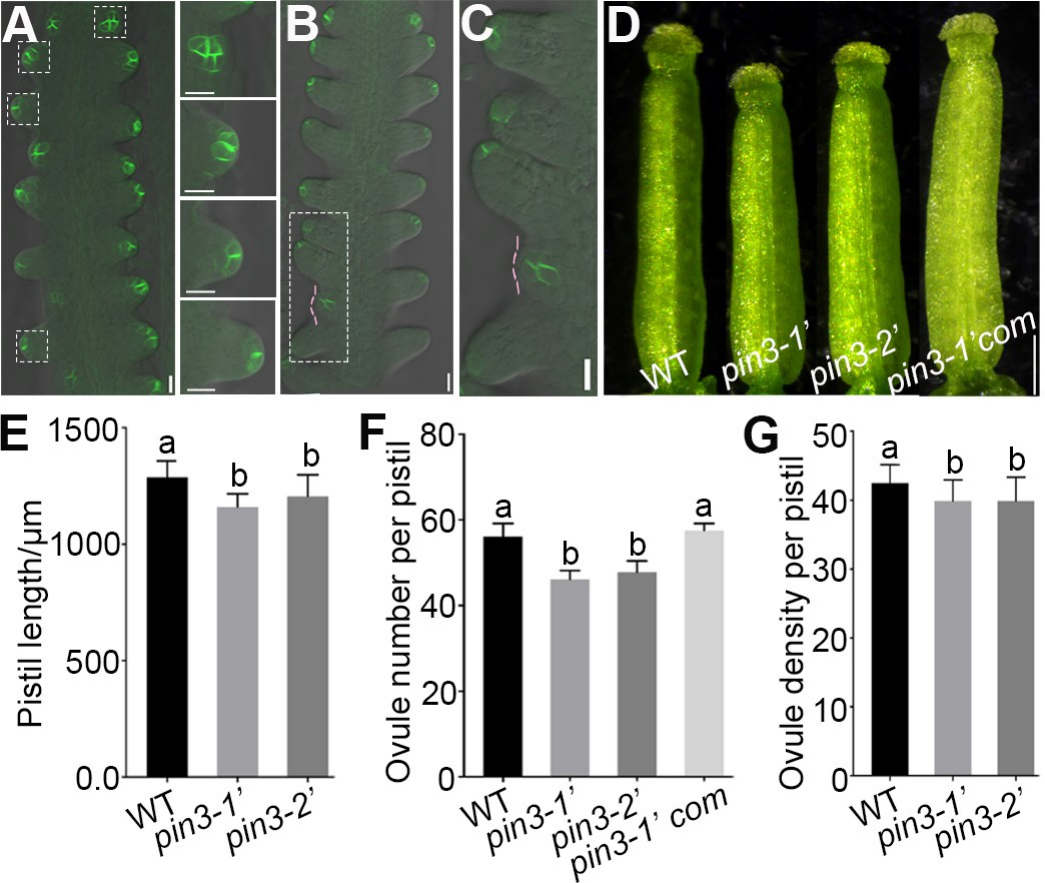
*PIN3* functions in ovule primordia initiation. (A) PIN3 protein localization during ovule primordia initiation in *pPIN3*::*PIN3-GFP* line. Magnified views of the dashed squares from top to bottom in left panel are shown in right panel. (B-C) PIN3-GFP is detected in the young ovule primordia (stage 1-I) at floral stage 10. Magnified view indicated by dashed rectangle in (B) is shown in (C). Pink dashed lines represent the young ovule primordia. (D) Pistil image of the wild-type, *pin3* mutants and the complementation line *pPIN3*::*PIN3-GFP pin3-1’ (pin3-1’com*). (E–G) The pistil length (E), ovule number per pistil (F), and ovule density (G) of the wild-type, *pin3* mutants and *pin3-1’com*. Data are presented as the mean ± SD (n = 21). Lowercase letters indicate significant differences revealed by one-way ANOVA (P < 0.01). Bars: 10 μm (A-C) and 250 μm (D).

We have demonstrated that *PIN3* positively regulates the seed number per silique and seed density in *A*. *thaliana* [49]. However, how *PIN3* controls seed number remains unclear. To determine the function of *PIN3* in ovule initiation and ovule number regulation, we first examined its expression pattern during early ovule developmental stages. Strong *pPIN3*::*PIN3-GFP* [50] expression was first detected in some cell clusters along the placenta at late floral stage 8, marking the initiation sites of ovule primordia (S1A and S1B Fig), which is consistent to previous study [4]. After the ovule primordia protruded, PIN3 localization gradually shifted toward the epidermal cells of the ovule primordia tip (stage 1-II) (Fig 2A), which is in agreement with prior report [43]. Most importantly, PIN3 was also detected in the new ovule primordia and the medial region of the pistil at floral stage 10 (Figs 2B, 2C and S1C and S1D), implying it participates in regulation of ovule initiation and pistil growth.

To unravel the importance of PIN3 in ovule initiation, we identified two new *pin3* mutants SALK_113246 and SALK_126753 and named them as *pin3-1* and *pin3-2*, respectively [49], which have the same names with the two reported *pin3* mutants [45]. These mutants are four different alleles. To distinguish the new alleles from existing *pin3-1* and *pin3-2* [45], we renamed *pin3-1* and *pin3-2* mentioned in our previous work [49] as *pin3-1*′ and *pin3-2*′ for this study. Compared with the wild-type, both two mutants had shortened pistils, reduced ovule number and density at floral stage 12 (Fig 2D–2G), which were not caused by poor nutrition as the *pin3* mutants exhibited normal vegetative growth and development (S1E Fig). Additionally, expression of *pPIN3*:*PIN3-GFP* in *pin3-1’* background (*pin3-1’com*) [49] could complement the defective phenotypes (Fig 2D and 2F), suggesting *PIN3* directly regulates ovule initiation and pistil growth.

### Mutations of *PIN3* disrupted polar auxin transport and decreased auxin response

Earlier research demonstrated that *PIN3* encodes an auxin efflux carrier that mediates the polar auxin transport essential for root growth and gravitropism [45]. Therefore, the gravitropism and auxin response were investigated. The bending angle of *pin3* mutants root tip was compromised upon gravi-stimulus (S2A and S2B Fig), indicating root gravitropism was impaired. Moreover, the root and hypocotyl length of *pin3* mutants were substantially shorter than those of the wild-type (S2A, S2C and S2E Fig). Further investigations revealed that the defective elongation of the hypocotyl epidermal cells was responsible for the decreased hypocotyl length of the mutants (S2D–S2F Fig). These auxin-related phenotypes of *pin3-1*′ and *pin3-2*′ are in accordance with *pin3-1* and *pin3-2* mutants identified in previous study [45], demonstrating that *pin3-1*′ and *pin3-2*′ were knock-down mutants of *PIN3*. Furthermore, the reduced elongation of the wild-type hypocotyl epidermal cells induced by the application of exogenous 1-naphthylphthalamic acid (NPA), which is an inhibitor of polar auxin transport, were similar to those of *pin3* mutant cells (S2D and S2F Fig), providing evidence that polar auxin efflux of *pin3* mutants was disrupted. Considered together, these results verified that knocking down of *PIN3* leads to the disrupted polar auxin transport and decreased auxin response.

### *PIN3* mainly regulates the late initiation of ovule primordia

**Fig 3:**
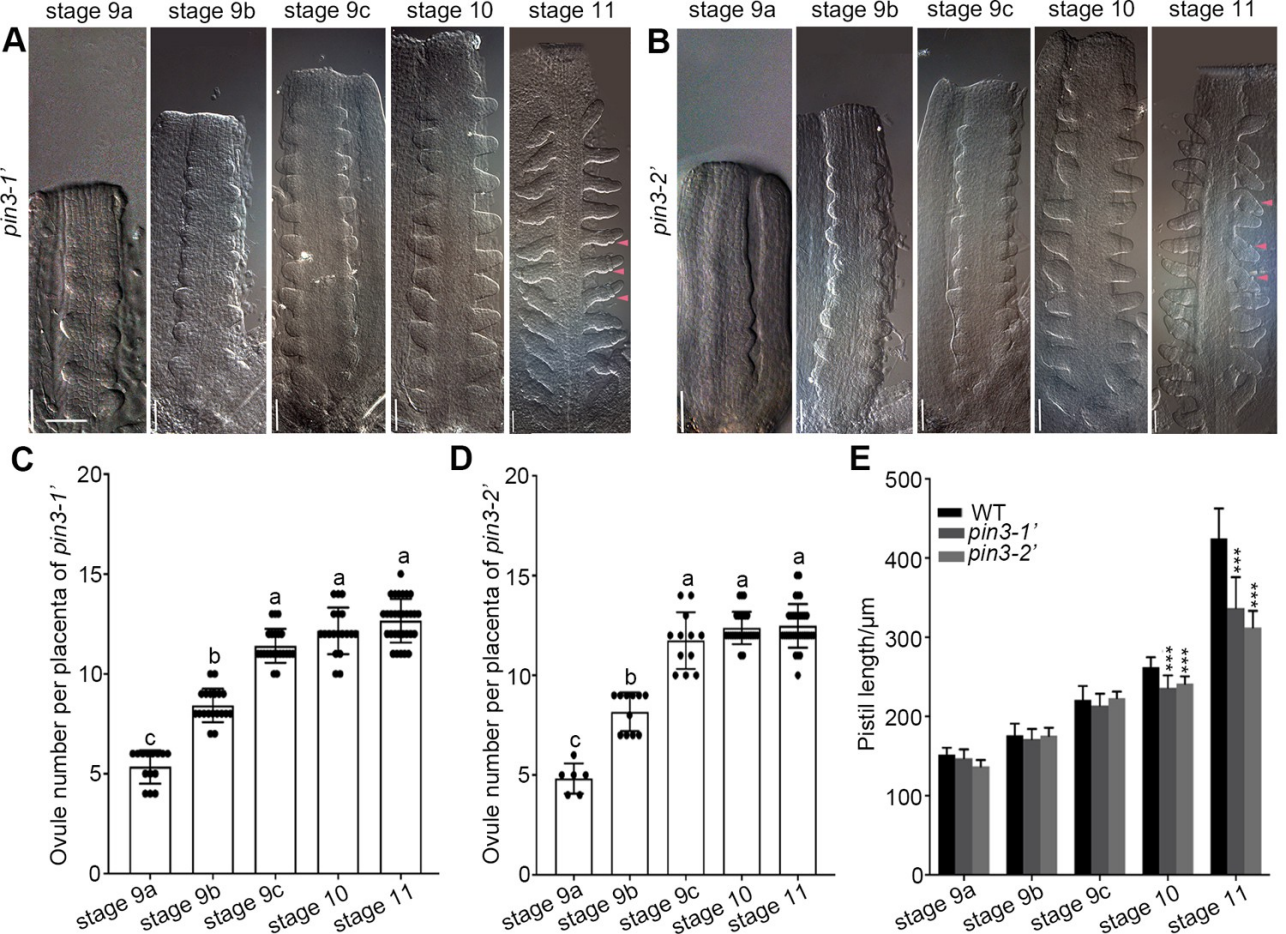
*pin3* mutants exhibite defective late ovule initiation. (A-B) Ovule primordia initiation process at floral stages 9a–11 in *pin3-1′* (A) and *pin3-2′* (B) mutants revealed by DIC. Pink arrows indicate the integument primordia. Bars: 20 μm. (C-D) Ovule number per placenta in *pin3-1′* (C) and *pin3-2′* (D). (E) Pistil length of the wild-type and *pin3* mutants. Data are presented as the mean ± SD, n = 15 (C-D) and 10 (E). Significant differences were tested by one-way ANOVA and showed in (C-D, lowercase letters, P<0.05) and in (E, *** P<0.001).

To characterize the *PIN3* function in the initiation of ovule primordia, we examined the ovule initiation process of *pin3* mutants at various floral stages. The ovule primordia protruded at floral stages 9a-c and developed into finger-shaped ovules (stage 2-I) at floral stage 10 in *pin3* mutants (Fig 3A and 3B). The ovule number per placenta increased significantly and then remained relatively stable at floral stage 10 (Fig 3C and 3D), which was in agreement with the observations of the wild-type (Fig 1B and 1C). At floral stage 11, we observed the integument primordia in most of the wild-type ovules at stage 2-III (pink arrows in Fig 1B) as well as a few young ovules at stage 2-I (yellow arrows in Fig 1B). However, all of the *pin3* ovules (stage 2-III) at this floral stage were similar regarding the size and shape accompanied by the integument primordia initiation, but young ovules at stage 2-I were undetectable (Fig 3A and 3B). Moreover, the ovule number of *pin3* mutants did not differ significantly between floral stages 10 and 11 (Fig 3C and 3D), further indicating there was no late ovule initiation at floral stage 10 in the absence of a functional *PIN3*. Besides, the pistils of *pin3* mutants were much shorter than that of the wild-type at floral stages 10 and 11 (Fig 3E). These results suggest that *PIN3* is essential for the late ovule initiation process.

### Disruption of *PIN3* leads to the compromised auxin signaling during ovule initiation

**Fig 4:**
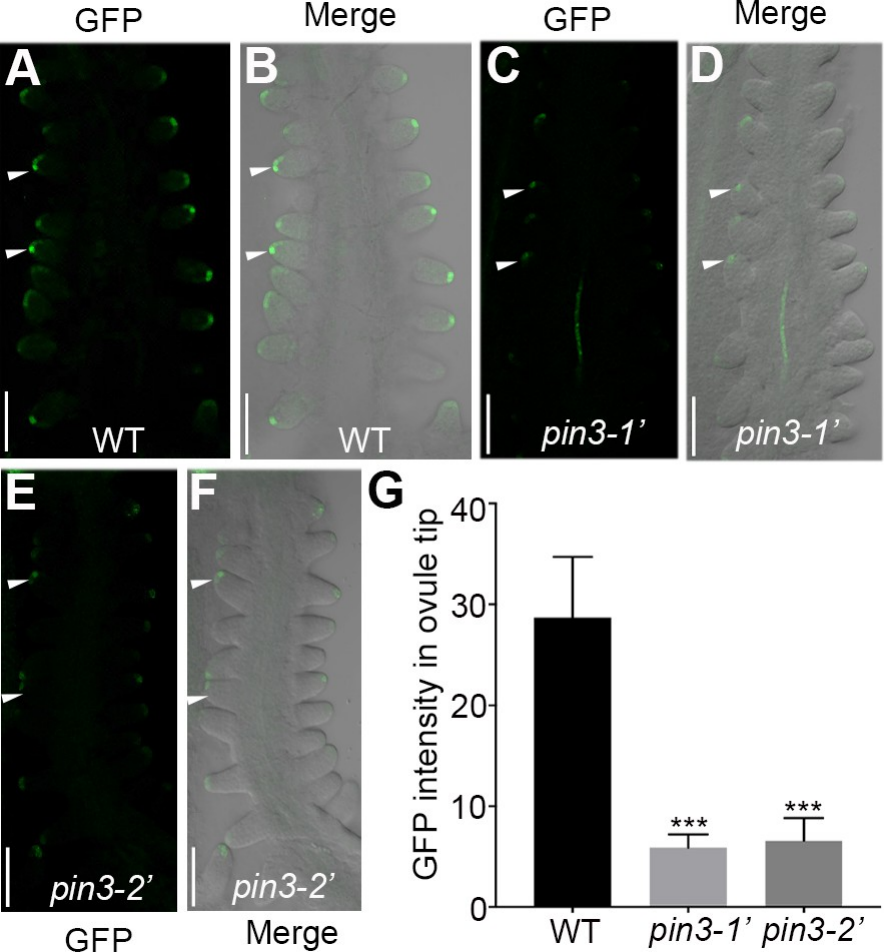
DR5 signal is compromised in *pin3* ovules at floral stage 10. (A-F) Confocal microscopy observations of the *DR5*::*NLS-eGFP* reporter in the wild-type (A-B), *pin3-1’* (C-D), and *pin3-2’* (E-F) backgrounds. (G) Quantification of DR5 fluorescence intensity in the ovule tip. Ten independent pistils were analysed. Bars: 50 μm. White arrows indicate the representative ovules for fluorescence intensity analysis. Data are presented as the mean ± SD. Significant differences were revealed by one-way ANOVA (*** P < 0.001).

DR5 is an auxin responsive element [51]. Its expression is induced by auxin and displays the cells with active auxin response [52]. DR5-NLS-eGFP has been widely used as an auxin responsive reporter [53], which provides a convenient tool to indicate the cells with auxin signaling in developing ovules [43]. To investigate whether the decreased ovule number of *pin3* mutants was due to the reduced auxin signaling during ovule initiation, we separately crossed the *pin3-1′* and *pin3-2′* mutants with the *DR5*::*NLS-eGFP* reporter line [53]. The fluorescence observations revealed that the DR5 signal in ovule tips at floral stage 10 was obviously weaker in *pin3-1′* and *pin3-2′* backgrounds than that in the wild-type (Fig 4A–4F). And the analysis of the fluorescence intensity showed that DR5 signal decreased dramatically in both *pin3* mutants (Fig 4G), indicating the auxin signaling decreased in the ovule tips probably because that less auxin was transported to the ovule tips in *pin3* mutants. These results reflected the importance of *PIN3* for the polar auxin transport required to establish auxin maximal which is sufficient for the late ovule initiation.

### Application of exogenous auxin analogue affects ovule initiation and its number

**Fig 5:**
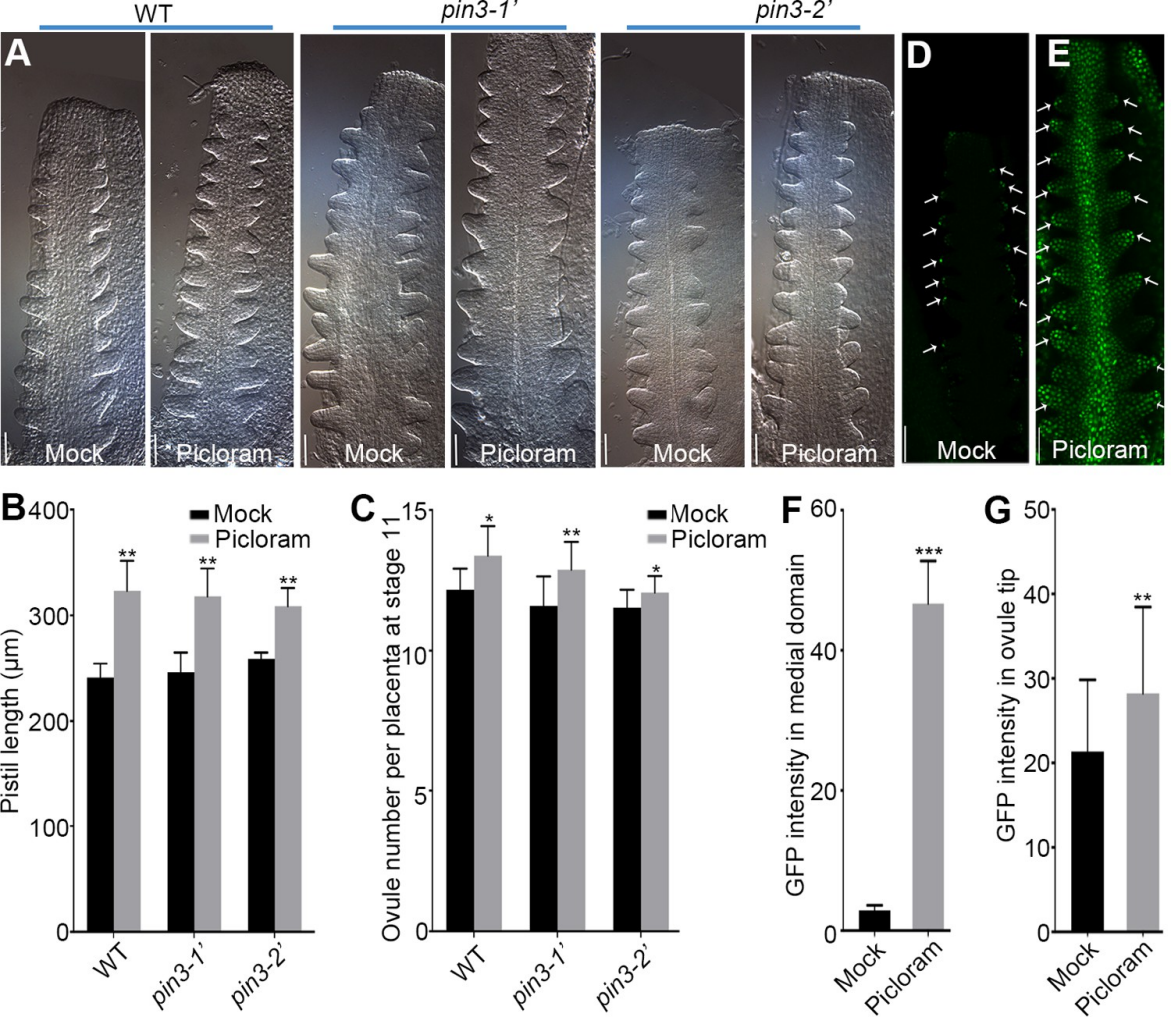
The picloram treatment recovers the ovule number of *pin3* mutants by altering auxin signal. (A) DIC images of the ovule initiation at floral stage 9c. Flowers grew for 2 days following the mock solution or 5 μM picloram treatment for 24 h. (B) Pistil length at floral stage 9c under picloram treatment. (C) Ovule number per placenta at floral stage 11. (D-E) Expression pattern of *DR5*::*NLS-eGFP* under the treatments of mock solution (D) or 5 μM picloram (E). White arrows indicate the ovule tips with GFP fluorescence. (F-G) GFP fluorescence intensity in the medial domain of the pistil (F) and ovule tip (G). Bars: 20 μm (A) and 50 μm (D-E). Data are presented as the mean ± SD (n = 12). Significant differences were revealed by one-way ANOVA (* P < 0.05, ** P < 0.01, *** P < 0.001).

To demonstrate that PIN3 regulates the late ovule initiation through enhancing auxin signal, we first treated the wild-type and *pin3* mutants with exogenous 1-naphthlcetic acid (NAA), an efflux substrate. This auxin analogue enters cells by passive diffusion and its accumulation level is dependent on the efflux carriers such as PIN proteins [54]. Statistical analysis showed that the ovule number per placenta of the wild-type and *pin3* mutants at floral stages 9c–11 wasn’t obviously increased under NAA treatment, implying that exogenous NAA did not efficiently enhance the auxin signal that is sufficient to promote ovule initiation, regardless of the presence of a functional *PIN3* in the wild-type (S3A–S3C Fig). Meanwhile, another auxin analogue, picloram was also applied to the wild-type and *pin3* mutants. Picloram is transported into cells by a special native plasma membrane-bound influx carrier PIC30, a member of the major facilitator superfamily (MFS) [55,56]. Interestingly, the pistil length and ovule number of the wild-type and *pin3* mutants were both significantly increased upon picloram treatment (Fig 5A–5C). Taken together, the picloram promotes ovule initiation and recovers the ovule number of *pin3* mutants, providing additional evidence that the decreased ovule number in *pin3* mutants may be caused by a lack of PIN3-mediated polar auxin transport.

### Picloram alters the local auxin distribution in the ovule and medial region of the pistil

To clarify how picloram promotes ovule initiation, we analyzed whether auxin signaling was affected upon picloram treatment. In the absence of picloram, the DR5::NLS-eGFP signal was mainly restricted to the ovule tips in agreement with the published data [11,28], indicating the maximal of auxin signal (Fig 5D). By contrast, the expression pattern of *DR5*::*NLS-eGFP* was interfered in the presence of picloram, resulting in a strong signal in the entire ovule and the medial region of the pistil (Fig 5E–5G). Therefore, the considerable auxin accumulation associated with the enhanced expression of *DR5*::*NLS-eGFP* in the medial region may explain the elongated pistil and increased ovule number in *pin3* mutants upon picloram treatment.

Several studies have demonstrated that *PIN1* encodes a major auxin transporter that regulates ovule initiation [11,28,33,47]. Therefore, we examined whether PIN1 protein levels are affected in *pin3* mutants. The western-blot result revealed that there is no significant difference of PIN1 protein levels between *pin3* mutants and the wild-type (S4 Fig), indicating that the normal PIN1 protein level could not cover the phenotype of no late ovule initiation caused by knocking down of PIN3. Therefore, we deduced that PIN3 may be the main player in the late ovule initiation.

### *STK* involves in PIN3-mediated late ovule initiation

**Fig 6:**
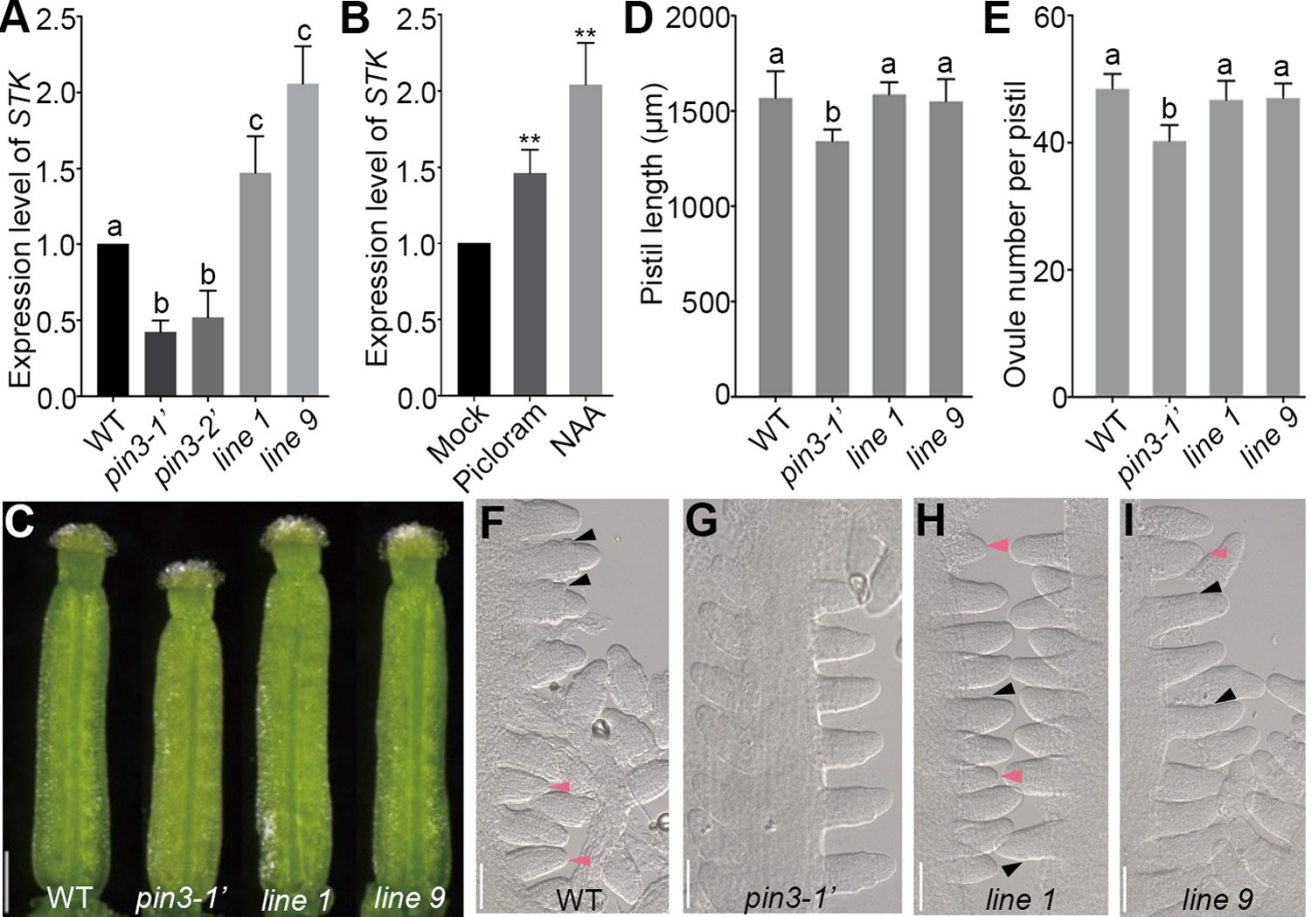
Overexpressing *STK* rescues the defective late ovule initiation of *pin3-1’* mutant. (A) qRT-PCR analysis of *STK* expression in the pistils of the wild-type, *pin3* mutants and *pSTK*::*STK-GFP pin3-1’* transgenic plants (*line 1* and *line 9*). (B) *STK* expression in the wild-type pistils was induced under 50 μM picloram and 100 μM NAA treatment for 1 h, respectively. The mock solution was used as a control. Three biological repeats were performed. (C) Image of pistils at floral stage 12. (D-E) Statistical assays of the pistil length (D), and ovule number per pistil (E). (F-I) DIC images showing the young ovules at floral stage 11 in the wild-type (F), *pin3-1’* (no young ovules) (G), *line 1* (H), and *line 9* (I). Pink and black arrows represent the young ovules and integument primordia, respectively. Bars: 250 μm (C) and 50 μm (F-I). Data are mean ± SD. Different lowercase letters indicate the significant differences tested by one-way ANOVA. P<0.05 (A), **P<0.01(B), P<0.01 (D) and P<0.001 (E).

To further investigate how *PIN3* affects the late ovule initiation, we examined the expression levels of several key genes related to the ovule identity-related genes, including *STK*, *ANT*, *AP2* and *SHP1*, and ovule boundary-related genes, including *ERL1*, *CUC1*, *CUC2* and *CUC3*. Interestingly, the expression level of *STK* was lower in *pin3* mutants than that in the wild-type while other genes didn’t significantly change (Figs 6A and S5A–S5G), which prompted us to test whether *STK* transcription responds to auxin. We performed qRT-PCR using the wild-type pistils which were collected from the treated inflorescences with auxin analogues (picloram and NAA). The results showed a dramatically up-regulation of *STK* expression by about 1.5 (picloram treatment) and 2.1 (NAA treatment) fold changes (Fig 6B), implying that *STK* may involve in PIN3-mediated late ovule initiation. To further explore the role of *STK* in this process, we introduced the *pSTK*::*STK-GFP* construction into *pin3-1’* mutant. Statistical analysis revealed that the decreased pistil length and ovule number were almost rescued by recovered expression of *STK* in *pin3-1’* mutant (Figs 6A, 6C–6E and S5H). Consistent with these results, DIC observations showed the young ovules (stage 2-I) at floral stage 11 in *pSTK*::*STK-GFP pin3-1’* plants (Fig 6F–6I), indicating that *STK* participates in PIN3-mediated late ovule initiation. Taken together, *PIN3* regulates the late ovule initiation and pistil growth probably through influencing *STK* function, as well as polar auxin transport and auxin response.

### BR regulates auxin response in the ovule tip and *PIN3* expression during ovule initiation

**Fig 7:**
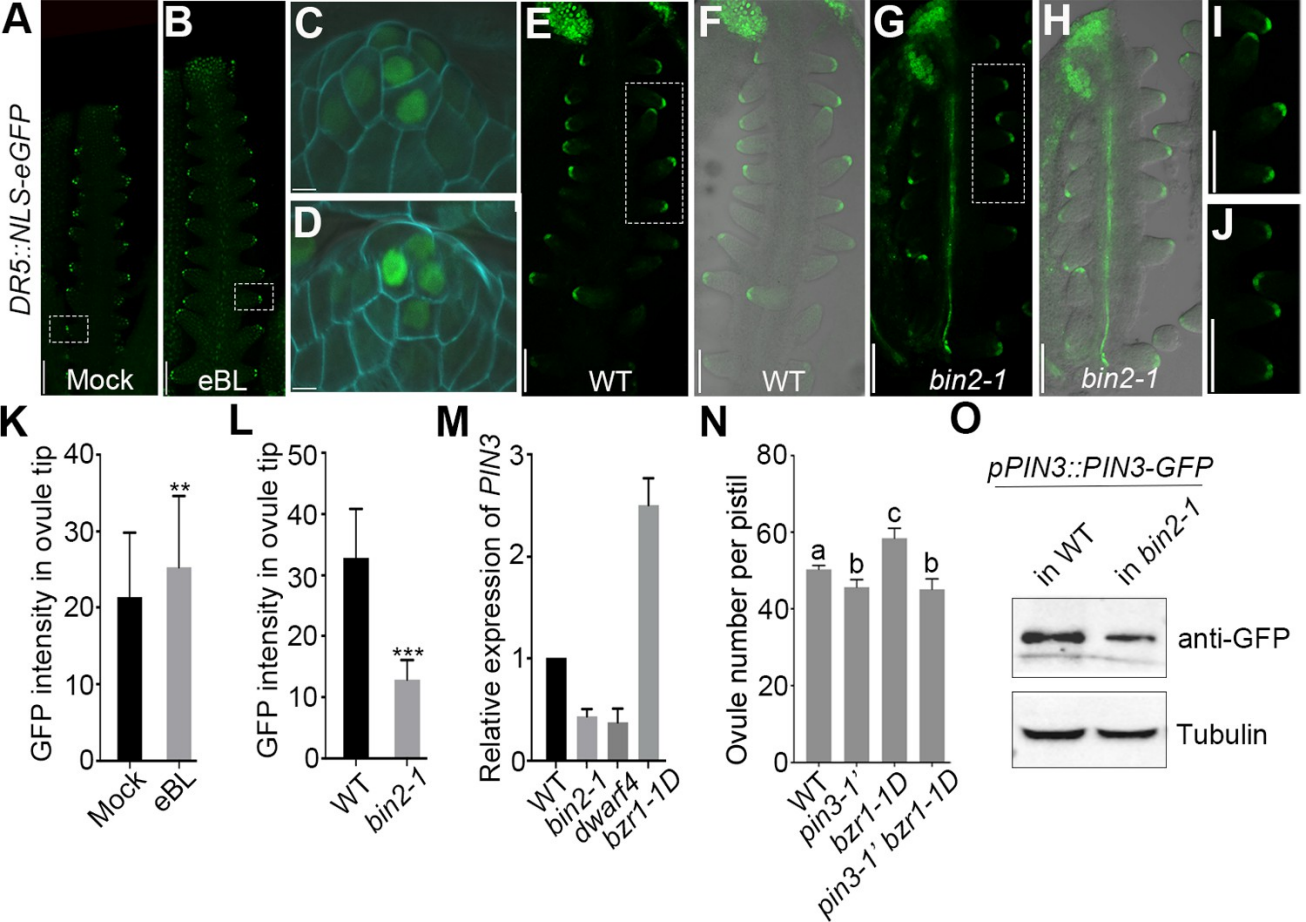
BR enhances auxin signal in the ovule tip and affects *PIN3* expression during ovule initiation. (A-D) Increased DR5 signal in ovule tips at floral stage 9 detected in *DR5*::*NLS-eGFP* line upon BR treatment. Magnified views of the ovules in (A) and (B) indicated by the dotted rectangle are shown in (C) and (D), respectively. Flowers were treated with the mock solution (A) or 2 μM eBL (B) for 24 h. (E-J) DR5 signal in ovule tips at floral stage 10 of the wild-type (E,F,I) and *bin2-1* (G,H,J) backgrounds. Magnified views of the ovules in (E) and (G) outlined by the dotted rectangle are shown in (I) and (J), respectively. (K-L) Quantification of GFP intensity in ovule tips under BR treatment (K) and in *bin2-1* mutant (L). (M) Expression analysis of *PIN3* in BR-related mutants. Three biological experiments were performed with similar results showing a time data. (N) Ovule number per pistil in the wild-type, *pin3-1’*, *bzr1-1D* and *pin3-1’ bzr1-1D* plants. (O) PIN3-GFP protein levels in the pistils of the wild-type and *bin2-1* mutant. Western blot was performed using anti-GFP antibody. Tubulin was severed as an internal control. Bars: 50 μm (A-J). Data are presented as the mean ± SD (n = 20). Significant differences were revealed by one-way ANOVA (** P < 0.01, *** P < 0.001). Different lowercase letters in (N) indicate the significant differences (P < 0.01).

**Fig 8:**
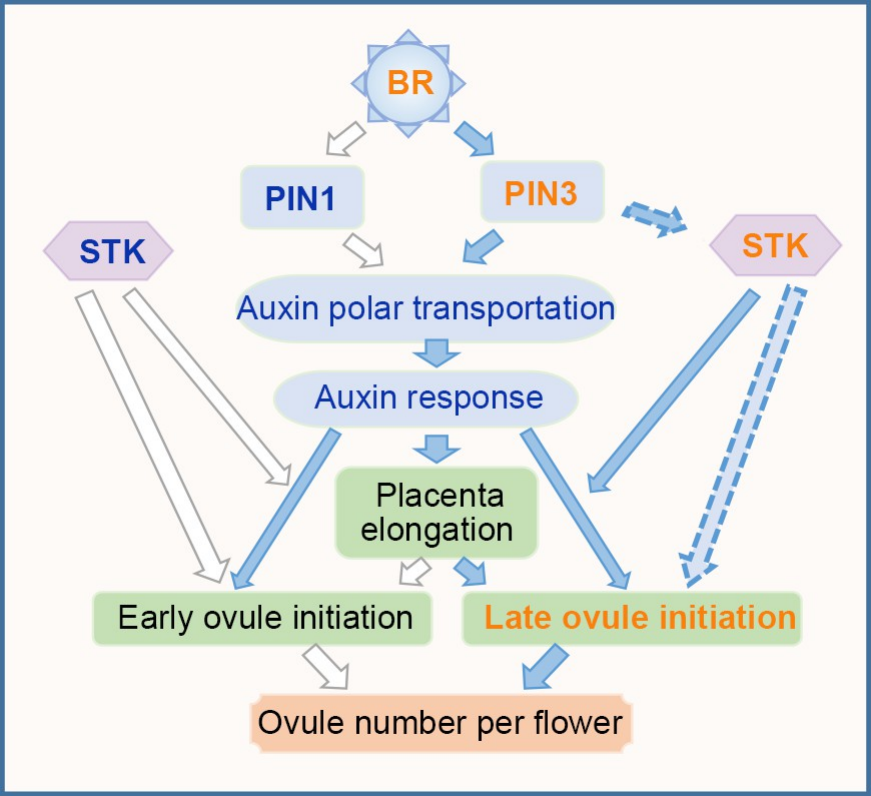
Proposed model for the ovule number determination in Arabidopsis. Ovule number per flower is determined by the early and late ovule initiation processes, while the early ovule initiation process has been demonstrated previously that partially controlled by BR-PIN1 pathway. Here, we discovered the late ovule initiation process mainly through BR-PIN3 pathway. BR positively regulates *PIN3* expression which in turn is required for auxin polar transport to form an auxin maxima in the placenta for ovule primordia initiation. Moreover, *PIN3* indirectly regulates *STK* expression, which is required for pistil growth and ovule identity, to promote pistil elongation providing enough space for the late ovule initiation.

It has been well studied that BR and auxin crosstalk and function in multiple developmental processes [57]. However, their integration in ovule initiation remains unclear. Prior studies demonstrated that the ovule number is decreased in BR-signal-reduced mutants, such as *bin2-1*, whereas increased in BR-signal-enhanced mutant *bzr1-1D* [39]. Moreover, *BIN2* participates in both BR and auxin signaling pathways [58,59]. Thus, to assess whether auxin signaling is involved in BR-mediated changes to the ovule number, we treated *DR5*::*NLS-eGFP* reporter plants with exogenous epi-brassinolide (eBL), which is an active form of BR. The DR5 signal and fluorescence intensity in the ovule tips were obviously stronger upon eBL treatment (Fig 7A–7D and 7K). While the DR5 signal and fluorescence intensity were visibly weaker in *bin2-1* mutant (Fig 7E–7J and 7L), indicating BR positively controls auxin signaling during ovule initiation. However, the applications of exogenous eBL failed to increase the pistil size and ovule number of *pin3* to the wild-type level (S6A–S6E Fig). More investigations revealed that the expression of *PIN3* was down-regulated in BR-signal-reduced mutants, *bin2-1* and *dwarf4*, and up-regulated in BR-signal-enhanced mutant, *bzr1-1D* (Fig 7M). Meanwhile, PIN3-GFP protein abundance in pistils was obviously reduced in *bin2-1* background compared to that of the wild-type plant (Fig 7O), which is consistent with the dramatically reduced GFP signal in the pistils of *pPIN3*::*PIN3-GFP bin2-1* plants at floral stage 9 (S7A–S7D Fig). In addition, PIN3-GFP signal in the root tip of *bin2-1* was also compromised compared to the wild-type, whereas PIN3-GFP localization did not show obvious abnormality (S7E–S7H Fig). Taken together, these results implied that BR signal positively regulates *PIN3* expression (at transcription level and protein level) but doesn’t impact PIN3 protein localization. What’s more, the ovule number of *bzr1-1D pin3* double mutant was close to that of *pin3* mutant (Fig 7N), suggesting that PIN3 is required for BR-mediated ovule initiation. Taken together, these results implied that PIN3 might function downstream of BR to regulate the ovule initiation. Our study results in a proposed model for BR-PIN3-STK regulation of ovule initiation that has been raised based on above data (Fig 8). Ovule initiation has early process and late process. PIN3-mediated auxin polar transport and response regulate the late process of ovule initiation.

## Discussion

### The multiple-step ovule initiation is important for the offspring number of Arabidopsis and seed yield of leguminous and cruciferous crops

The previous study indicated that the early ovule initiation ceased at floral stage 10 and all existing ovules started to differentiate [2,11]. However, the ovule number at this stage represents only 90% of the total number of ovule primordia, indicative of the late ovule initiation beyond early ovule initiation. In this study, the significantly increased ovule number at floral stage 11 compared with floral stage 10 and the expression of *pPIN1*::*PIN1-GFP* reporter further demonstrated that some young ovule primordia formed after the early ovule initiation (Fig 1). However, only around 10% ovule primordia were generated in the late process. Because the young ovules protruded merely from some long boundary regions, we hypothesized that the late ovule initiation was due to the considerable available space, and nutrients which promoted the late initiation through expanding the placenta size. Above all, there are multiple steps in the intact process of ovule initiation and all steps contribute to the total number of ovule primordia, including the two groups of ovule primordia produced during the early ovule initiation process and the sporadic ovule primordia generated during the late ovule initiation process. This complicated process may have biological significance. We speculate that the multiple steps of ovule initiation might be an adaptive strategy during plant evolution to sequentially promote ovule initiation to fit the space or nutrient supply for maximum production of offspring. In addition, different ecotypes and ages [6], environmental factors such as heat stress [60], also have great impact on ovule number. We deduce that these factors probably affect the early and late initiation of ovule primordia. The regulatory mechanism of multiple- step ovule initiation would be useful to increase seed number and yield of leguminous and cruciferous crops, which also have multiple ovules in one placenta/pistil.

### Polar auxin transport and auxin signaling are essential for the late ovule initiation

The polar localization of PIN3 is required for the auxin efflux-dependent asymmetrical distribution of auxin and root gravitropism [45]. Consistent with the reported data, the *pin3-1′* and *pin3-2′* mutants exhibited auxin-related defective phenotypes, including compromised gravitropic response, decreased length of root and hypocotyl epidermal cells (S2 Fig). Hence, knocking down of PIN3 adversely affects polar auxin transport and auxin response.

Previous studies and our results revealed that DR5 signal is difficult to detect in placenta but easy to detect in ovule tips after ovule primordia protrusion (Figs 5D and 7A) [11,28], thus its expression in the ovule tips can be an indicator to mark the regions with active auxin signal. In *pin3* mutants, compromised auxin signal in the ovule tips are revealed by the decreased intensity of DR5 signal (Fig 4), implying that the formation of effective auxin maxima is affected during the late ovule initiation. Consistently, the efficient treatment of NAA is controlled by the efflux carriers including PIN3 [54]. However, knocking down of PIN3 leads to disturbed auxin flow and fails to form the effective auxin maximal required for the late ovule initiation even in the presence of NAA (S3 Fig). By contrast, picloram, a synthetic auxin analogue, is absorbed rapidly by a special influx transporter PIC30 and accumulates in growing tissues [55,56]. Thus, the rapid uptake and accumulation of picloram, which is independent with PIN3, may be liable to enhance total auxin signaling and response (Fig 5D–5G). It might be the main reason for picloram promotion of pistil growth and ovule initiation in the wild-type and *pin3* mutants (Fig 5A–5C). Taken together, the application of NAA could not effectively restore the defective phenotype of the late ovule initiation, while picloram could, further suggesting that the formation of auxin maximal mediated by PIN3 polar transport is essential for the late ovule initiation (Figs 5 and S3).

### PIN1 and PIN3 probably overlap in regulating the late ovule initiation

Previous studies and our results showed that the spatiotemporal expression of PIN3 overlaps with PIN1 in the medial domain/replum, lateral domain/valve, stigma, and style during early pistil (floral stages 5–8) and ovule (stages 1-I to 2-I) development mediating auxin flow (Figs 2A–2C and S1A and S1B) [2,4,43,46,61]. However, the detailed localizations of PIN1 and PIN3 are diverse. During ovule initiation, apolar PIN1 localization is initially observed in plasma membrane of placenta cells, and gradually shifts toward the transverse and lateral sides of several cell clusters, which develop to ovule primordia, to pump auxin toward the incipient ovule primordia tip (Fig 1D and 1E) [11,28,46,47]. Different from PIN1, strong *pPIN3-PIN3-GFP* is first detected in several cell clusters which develop to ovule primordia (S1A and S1B Fig) [4]. These results suggest that PIN3 probably expresses later than PIN1 and their localization overlaps in the apical portion of the ovule primordia. Therefore, we deduce that PIN1 and PIN3 may have redundant functions in the early ovule initiation, while PIN1 is the major player in the early process since the weak *pin1-5* mutant only has on average 9 ovules [11,28,33]. Whereas the *pin3* mutants have no defects in early process, which might be due to the comparable pistil length of *pin3* mutants at stages 9a-9c to the wild-type (Fig 3E). By contrast, in *pin3* mutants, PIN1 is not affected (S4 Fig), and the extra ovule number is specifically reduced at floral stage 10 which might be caused by the reduced pistil length at this stage (Figs 2F and 3A–3E), implying that enough space is critical for the late ovule initiation. Therefore, the functions of PIN1 and PIN3 may overlap in the late ovule initiation, while PIN3 is the main player in the late process. Besides, PIN3 may mainly regulate this process through affecting pistil growth to provide enough space for the late ovule initiation.

### Enough space is important for the late ovule initiation

Previous studies have revealed that the pistil size is usually correlated with the ovule number [6]. Our results demonstrate that *pin3* mutants displayed a reduction in pistil size as well as defective in late ovule initiation (Fig 3A–3E), indicating PIN3-dependent placenta space is tightly related with the late ovule initiation. Other evidences came from the ovule number recovery under picloram treatment (Fig 5A–5C) and after introduction of *pSTK*::*STK-GFP* in *pin3-1’* (Figs 6 and S5H), further suggesting that the reduced pistil size was the reason of lacking enough boundary regions which are required for the late ovule initiation. *STK* is a key regulator of developmental signals for ovule identity and pistil growth. Loss-of-function mutant *stk* has short pistil and less ovules [18]. *STK* expression largely overlaps with that of *PIN3* in the ovule primordia and medial domain of pistil [4,18]. *STK* is induced by auxin (Fig 6B) and overexpressing *STK* possibly mimics enhanced auxin signaling and promotes ovule initiation in *pin3* to rescue its ovule number phenotype, suggesting STK directly involves in PIN3-mediated auxin-related late ovule initiation. Or *STK* and *PIN3* co-contributes to the process in different ways. *STK* also could indirectly recover the late ovule initiation through promoting pistil growth. It seems that the developmental and auxin signals might be integrated in regulating pistil size and late ovule initiation in Arabidopsis. These findings further provide promising strategies of promoting pistil growth and ovule initiation in cruciferous and leguminous crops by manipulating related gene expression. Therefore, the connection between these two signal pathways is worth studying in the future to understand the regulatory networks affecting pistil size and ovule number.

### BR promotes ovule initiation by regulating *PIN3* expression

Our previous work demonstrated that BR positively regulates ovule number [39], and BR enhances auxin signal during ovule initiation [11]. In this study, we found that BR treatment enhances auxin response in the wild-type (Fig 7A–7D) but fails to promote the late ovule initiation and increase the ovule number in *pin3* mutants which results consistent with *bzr1-1D pin3-1’* double mutant (Figs 7K, 7N and S6), implying that PIN3-meditated auxin polar transport is required for BR promotion of ovule initiation. BR signal positively regulates PIN3 expression levels but does not affect PIN3 localization (Figs 7M, 7O and S7), further suggesting PIN3 works downstream of BR signal in ovule initiation. In agreement with above results, the BR-signal-reduced mutant *bin2-1* also displays compromised auxin response in the ovule tips (Fig 7E–7J and 7L), which might be caused by the significantly reduced expression of *PIN3* in the ovules (S7A–S7D Fig). Taken together, our results suggested that BR regulates ovule initiation partially through influencing *PIN3* expression.

Overall, we proposed a hypothetical model for BR-PIN3-STK regulation of ovule initiation. Ovule initiation has early process and late process. The dynamics of PIN1 polar localization, auxin polar transport and response regulate the early process of ovule initiation [11]. *PIN3* mediates the late process of ovule initiation, and BR promotes this process through regulating PIN3 expression. *STK* involves in PIN3-mediated late ovule initiation probably through promoting placenta elongation to provide enough space for the late ovule initiation (Fig 8). Our study describes the multiple steps and regulatory mechanism of ovule initiation, giving clues to increase seed number and yield of leguminous and cruciferous crops.

## Materials and methods

### Plant materials and growth conditions

The *PIN3* (AT1G70940) T-DNA insertion mutants SALK_113246 and SALK_126753, and *pPIN3*::*PIN3-GFP pin3-1’* complementation plant [49], the BR related mutants *bzr1-1D* [62], *dwarf4* [63] and *bin2-1* [59] in Col-0 background used in this study have been reported previously. The *DR5*::*NLS-eGFP* [53], *pPIN1*::*PIN1-GFP* [48], and *pPIN3*:*PIN3-GFP* [50] marker lines were also demonstrated in previous studies. Seeds were sterilized in 75% ethanol for 5 min, washed three times with sterile water, and then placed on half-strength Murashige & Skoog (1/2 MS) medium containing 1% sucrose and 0.7% agar (pH 5.8) in Petri plates. After a 2-day vernalization at 4°C, the plates were placed in an illumination incubator for 7 days. The seedlings were transferred to soil and grown at 22°C with a 16h light/8h dark cycles.

### Plasmid construction and plant transformation

For the *pSTK*::*STK-GFP* construction, about 2008 bp promoter fragments upstream of the *STK* translation start site were amplified using primers pSTK-F (containing *EcoR*I restriction site) and pSTK-R (containing *BamH*I restriction site). The obtained PCR product was cloned in the *EcoR*I and *BamH*I sites of a binary destination vector pHB backbone which fused with *GFP* reporter gene using homologous recombination kit (Vazyme). Then, the full-length genomic region containing 3779 bp was amplified with primers gSTK-F and gSTK-R (both containing *BamH*I restriction site). This PCR product was cloned in *BamH*I site of the *pHB-pSTK-GFP* intermediate vector as described above. This *pSTK*::*STK-GFP* construct was transformed into *Agrobacterium tumefaciens* (strain GV3101). Eventually, *pin3-1’* mutants were transformed using the floral dip method [64], and the transgenic plants were characterized by hygromycin screening. The primers used for cloning and genotyping are listed in S1 Table.

### qRT-PCR analysis

Total RNA was extracted from the pistils using TRIzol reagent (Invitrogen). The first-strand cDNA was synthesized using the FastKing RT Kit (with gDNase) (Tiangen). The SYBR Green Realtime PCR Master Mix (Toyobo) and the QuantStudio 3 Real-Time PCR system (Thermo Fisher) were used for the qRT-PCR running. *AtACTIN7* (AT5G09810) served as the internal control for normalizing relative gene expression levels. Three biological experiments were performed and each with three technical replicates. Details of qRT-PCR primers are listed in S1 Table.

### Western blot analysis

Total proteins were extracted using 2 x sodium dodecyl sulfate (SDS) buffer containing 0.5 M Tris·HCl (pH 6.8), 3% dithiothreitol (DTT), 4% (w/v) SDS, 20% (v/v) glycerol and 0.2% (w/v) bromophenol blue. Then, the protein samples were placed in the boiling water for 10 min following by on the ice. The mixture of proteins was separated on 12% SDS polyacrylamide gel electrophoresis (SDS-PAGE) and subsequently transferred onto a polyvinylidene fluoride (PVDF) membrane (Whatman, Buckinghamshire, UK). The membranes were incubated with the first antibodies anti-PIN1 (1:3000, Abiocode R2114-3), anti-GFP (1:5000, Abways) and internal control Tubulin (1:3000, Abiocode M0267-1a), respectively, overnight at 4°C after blocking with 5% skim milk. The blocked membranes were washed three times by phosphate buffered saline tween-20 (PBST) each step for 10 min. Then, the membranes were incubated with the secondary antibody Goat anti-Rabbit or Goat anti-Mouse IgG with HRP-conjugated (1:5000, Abiocode) for 3 h at room temperature and washed three times by PBST. The signals were showed by ECL solutions (Thermo Fisher) for 5 min in the darkness and were photographed by Bio-Rad machine with a charge couple device (CCD) camera system.

### Microscopy analysis

Regarding the DIC microscopy observation for the ovule primordia initiation process, the pistils of wild-type and mutant plants were isolated from the flower buds at stages 9a–11 and then immersed in the clearing agent comprising chloral hydrate, H_2_O, and glycerol (8:3:1) until the pistils cleared. The treated pistils were examined using the Zeiss Axio Imager M2 microscope with a DIC channel. The GFP signal in dissected pistils immersed in 50% glycerol was detected using the TCS SP8 confocal laser scanning microscope (Leica). The excitation and emission wavelengths were 488 nm and 500–560 nm, respectively. The fluorescence intensity was analyzed using the Image J software.

### Root gravitropism analysis

7-d-old seedlings of wild-type and *pin3* mutants incubated on vertical plates were orientated on horizontal to subjected to the gravity stimulation for indicated time and photographed. The length of root, hypocotyl and hypocotyl epidermis cells, and the bending angle of root tip were measured using the Image J software.

### Physiological assay

To examine the response of hypocotyl epidermis cells to 1-naphthylphthalamic acid (NPA; Sigma-Aldrich 399728), seedlings of wild-type were grown on 1/2 MS with or without 10 μM NPA for 7 days. The morphology of hypocotyl epidermis cells was observed by DIC.

To analyze the formation of ovule primordia upon 1-naphthylacetic acid (NAA; Sigma-Aldrich 317918), picloram (Sigma-Aldrich 1918021) and Epibrassinolide (eBL; Sigma-Aldrich 78821439) treatments, the floral buds larger than stage 10 were removed and the remaining inflorescences were immersed in 0.01% (v/v) Silwet L-77 solutions containing 2 μM eBL, 2 μM NAA, or 5 μM picloram as previously described [65]. The control inflorescences were treated with 0.01% Silwet L-77 solutions containing corresponding volume ethanol or DMSO. After 24 h, the inflorescences were washed with distilled water to remove residual chemicals. The pistils were collected 2 days later.

For qRT-PCR analysis, the whole wild-type inflorescences were immersed in the mock (containing 0.5% DMSO or 1% ethanol), 50 μM picloram and 100 μM NAA solutions, respectively, as described above. The pistils were collected after 1 h for later analysis.

## Supporting information

S1 Fig *PIN3* expression and phenotypic analysis of *pin3* mutants. (A-B) *PIN3* expresses in some cell clusters along the placenta which develop to the ovule primordia at late floral stage 8. (C-D) *PIN3* expresses in the medial region of the pistil at floral stage 10. White arrows indicate the ovule primordia (A-B) and the medial region (C-D). (E) Images of 6-week-old *pin3-1’* and *pin3-2’* plants showing normal growth and development to that of the wild-type. Bar: 10 μm (A-D) and 1 mm (E).
(TIF)

S2 Fig Phenotypic analysis of *pin3* mutants. (A) Representative images of root growth and gravimetric response. (B) Statistical analysis of root bending angle in the wild-type and *pin3* mutants at specific time-points under gravi-stimulus (n = 15). (C) Root length of the wild-type and *pin3* mutants (n = 15). (D) DIC images of the hypocotyl epidermal cells of the wild-type, NPA-treated wild-type, *pin3-1′* and *pin3-2′* plants. Black arrows indicate the cell boundaries. (E) Hypocotyl length of the wild-type and *pin3* mutants (n = 16). (F) Hypocotyl epidermal cell length shown in (D) (n = 34). Bars: 5 mm (A) and 50 μm (D). Data are presented as the mean ± SD. Significant differences were determined by one-way ANOVA (*** P < 0.001).
(TIF)

S3 Fig Exogenous application of NAA can’t rescue the late ovule initiation of *pin3* mutants. (A–C) Ovule number per placenta at floral stages 9c-11 in the wild-type (A), *pin3-1′* (B), and *pin3-2′* (C) plants. Flowers were immersed into the mock solution or 2 μM NAA for 24 h. Ovule number was recorded after 2 days following NAA treatment. Data are presented as the mean ± SD (n = 15). Lowercase letters indicate significant differences revealed by one-way ANOVA (P < 0.01). There were no significant differences between the mock control and NAA treatment.
(TIF)

S4 Fig Endogenous PIN1 protein level in the wild-type and *pin3* mutants. The level of PIN1 protein was detected with the anti-PIN1 antibody. Tubulin was used as an internal control. Three independent experiments were performed with similar results.
(TIF)

S5 Fig Expression analysis of the ovule identity and boundary related genes in *pin3* mutants. (A-G) qRT-PCR indicates the transcription levels of ovule identity-related genes *ANT* (A), *AP2* (B), *SHP1* (C), and boundary-related genes, *ERL1* (D), *CUC1* (E), *CUC2* (F) and *CUC3* (G), respectively. Pistils were collected from the wild-type and *pin3* mutants for this assay, which was completed with three biological replicates. (H) Semi-quantitative PCR analysis of exogenous *STK* expression in *pSTK*::*STK-GFP pin3-1’* transgenic plants. *AtACTIN 7* was used as an internal control.
(TIF)

S6 Fig eBL treatment can’t recover the pistil length and ovule number per placenta of *pin3* mutants. (A) The pistil length at floral stage 11 under eBL treatment. (B) The ovule number per placenta at floral stage 11. (C-E) Ovule number per placenta in the wild-type (C), *pin3-1′* (D) and *pin3-2′* (E) plants at floral stages 9a-11. Pistils were collected at 2 days after 24 h treatment with the mock solution or 2 μM eBL. Data are presented as the mean ± SD (n = 20). Significant differences were tested by one-way ANOVA (** P < 0.01).
(TIF)

S7 Fig BR-signal-reduced mutant *bin2-1* affects the expression of *PIN3* but not the protein localization. (A-D) *pPIN3*::*PIN3-GFP* expression in the ovule primordia of the wild-type (A-B), and *bin2-1* mutant (C-D) at floral stage 9, respectively. White arrows represent the ovule primordia. (E-F) PIN3-GFP fused protein is uniformly localized in the columella cell boundaries of the wild-type root tip. (G-H) PIN3-GFP shows similar localization in the columella cell boundaries of the *bin2-1* root tip to that of the wild-type. Bars: 10 μm.
(TIF)

S1 Table Primers used for PCR in this study. (XLSX)
