# Supplementary figures and images for: PIN3 positively regulates the late initiation of ovule primordia in Arabidopsis thaliana
Source: PLoS Genet. 2022 Mar 4;18(3):e1010077. doi: 10.1371/journal.pgen.1010077 (PMC8896676; doi:10.1371/journal.pgen.1010077)

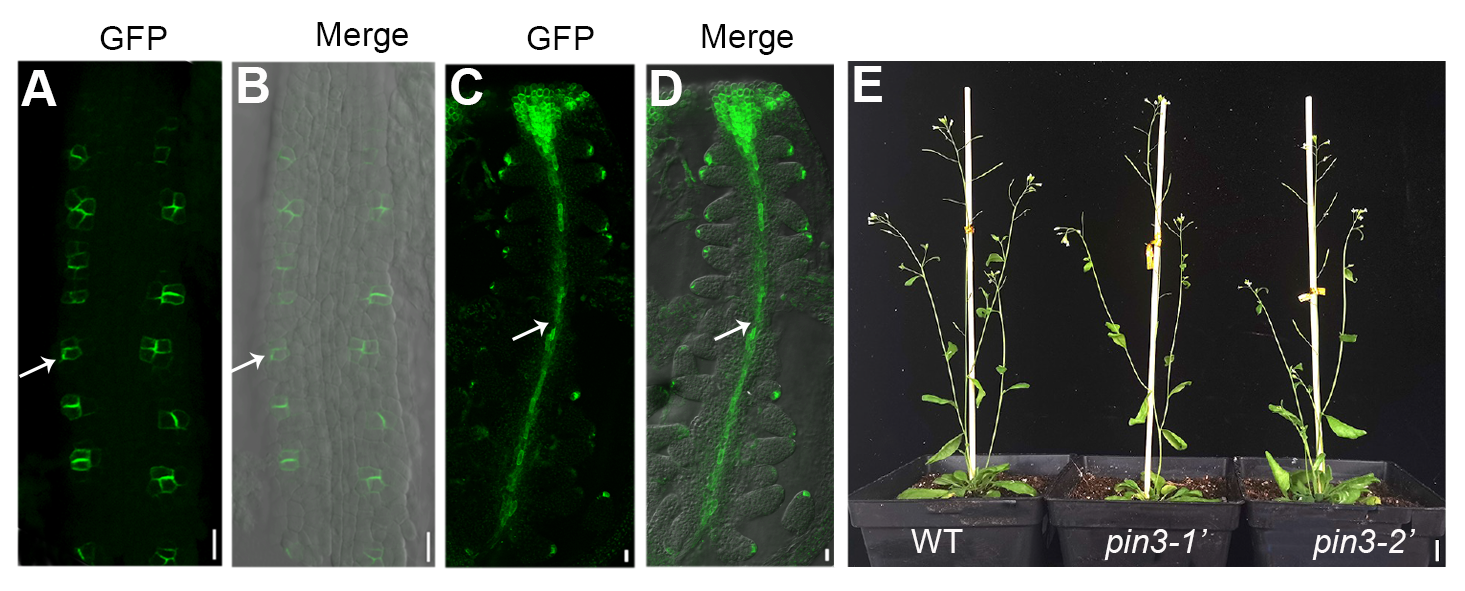

Supplement: S1 Fig — (A-B) PIN3 expresses in some cell clusters along the placenta which develop to the ovule primordia at late floral stage 8. (C-D) PIN3 expresses in the medial region of the pistil at floral stage 10. White arrows indicate the ovule primordia (A-B) and the medial region (C-D). (E) Images of 6-week-old pin3-1’ and pin3-2’ plants showing normal growth and development to that of the wild-type. Bar: 10 μm (A-D) and 1 mm (E). (TIF) [file pgen.1010077.s001.tif]

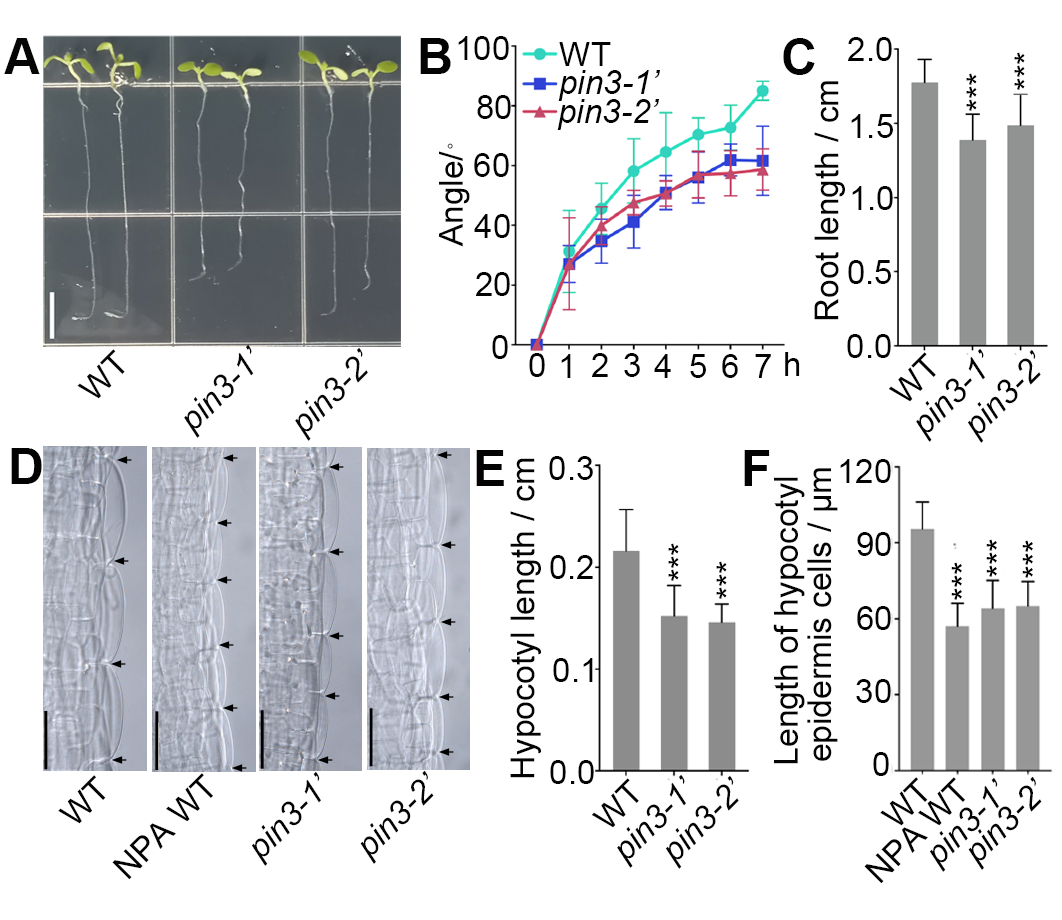

Supplement: S2 Fig — (A) Representative images of root growth and gravimetric response. (B) Statistical analysis of root bending angle in the wild-type and pin3 mutants at specific time-points under gravi-stimulus (n = 15). (C) Root length of the wild-type and pin3 mutants (n = 15). (D) DIC images of the hypocotyl epidermal cells of the wild-type, NPA-treated wild-type, pin3-1′ and pin3-2′ plants. Black arrows indicate the cell boundaries. (E) Hypocotyl length of the wild-type and pin3 mutants (n = 16). (F) Hypocotyl epidermal cell length shown in (D) (n = 34). Bars: 5 mm (A) and 50 μm (D). Data are presented as the mean ± SD. Significant differences were determined by one-way ANOVA (*** P < 0.001). (TIF) [file pgen.1010077.s002.tif]

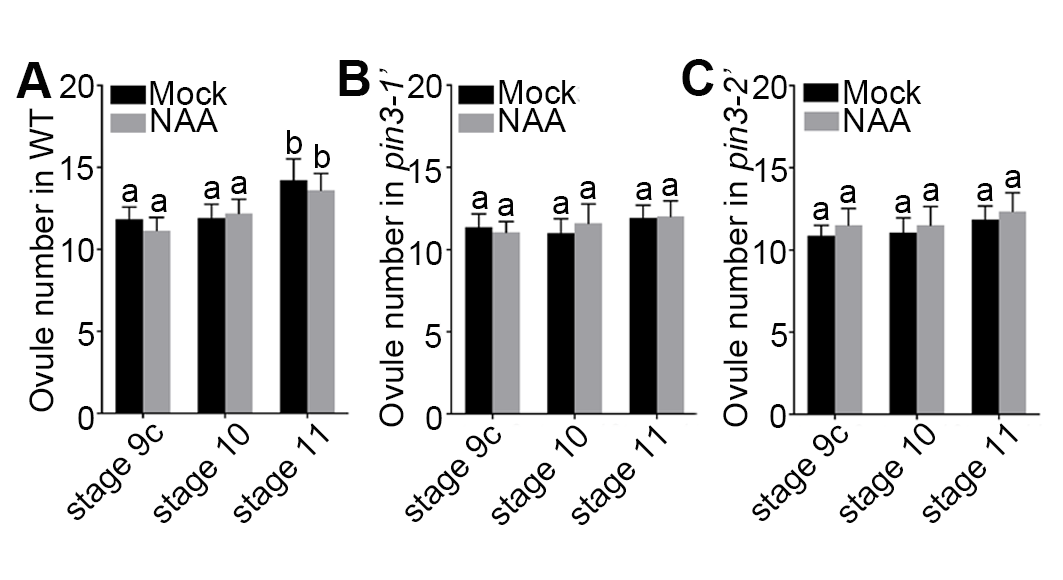

Supplement: S3 Fig — (A–C) Ovule number per placenta at floral stages 9c-11 in the wild-type (A), pin3-1′ (B), and pin3-2′ (C) plants. Flowers were immersed into the mock solution or 2 μM NAA for 24 h. Ovule number was recorded after 2 days following NAA treatment. Data are presented as the mean ± SD (n = 15). Lowercase letters indicate significant differences revealed by one-way ANOVA (P < 0.01). There were no significant differences between the mock control and NAA treatment. (TIF) [file pgen.1010077.s003.tif]

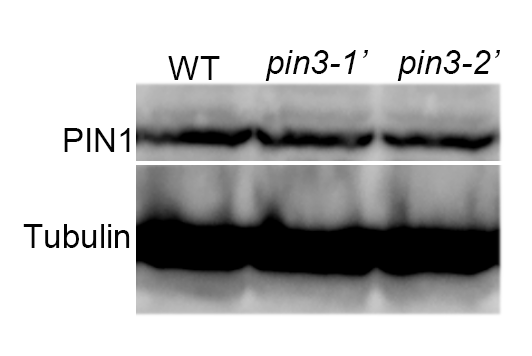

Supplement: S4 Fig — The level of PIN1 protein was detected with the anti-PIN1 antibody. Tubulin was used as an internal control. Three independent experiments were performed with similar results. (TIF) [file pgen.1010077.s004.tif]

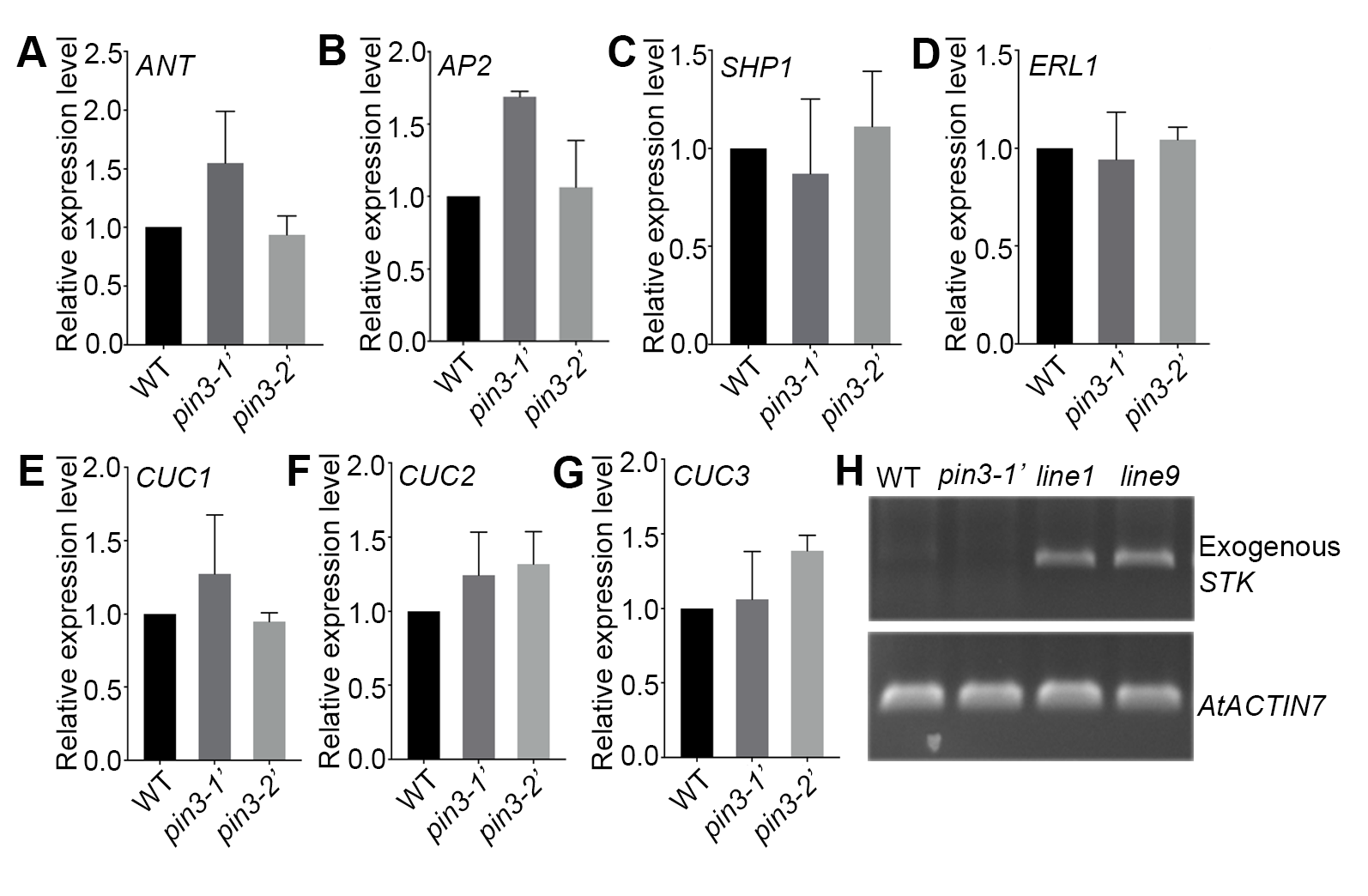

Supplement: S5 Fig — (A-G) qRT-PCR indicates the transcription levels of ovule identity-related genes ANT (A), AP2 (B), SHP1 (C), and boundary-related genes, ERL1 (D), CUC1 (E), CUC2 (F) and CUC3 (G), respectively. Pistils were collected from the wild-type and pin3 mutants for this assay, which was completed with three biological replicates. (H) Semi-quantitative PCR analysis of exogenous STK expression in pSTK::STK-GFP pin3-1’ transgenic plants. AtACTIN 7 was used as an internal control. (TIF) [file pgen.1010077.s005.tif]

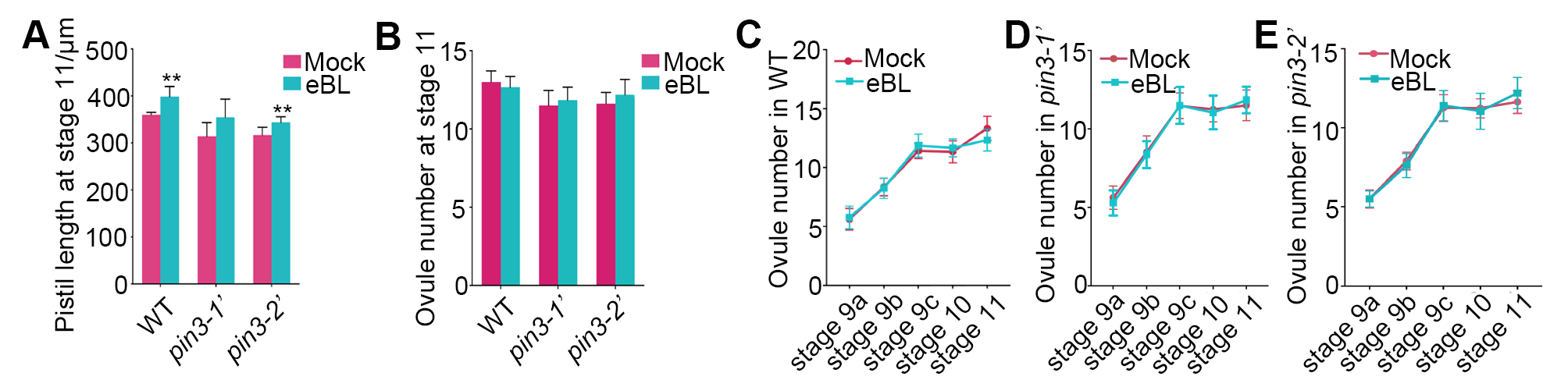

Supplement: S6 Fig — (A) The pistil length at floral stage 11 under eBL treatment. (B) The ovule number per placenta at floral stage 11. (C-E) Ovule number per placenta in the wild-type (C), pin3-1′ (D) and pin3-2′ (E) plants at floral stages 9a-11. Pistils were collected at 2 days after 24 h treatment with the mock solution or 2 μM eBL. Data are presented as the mean ± SD (n = 20). Significant differences were tested by one-way ANOVA (** P < 0.01). (TIF) [file pgen.1010077.s006.tif]

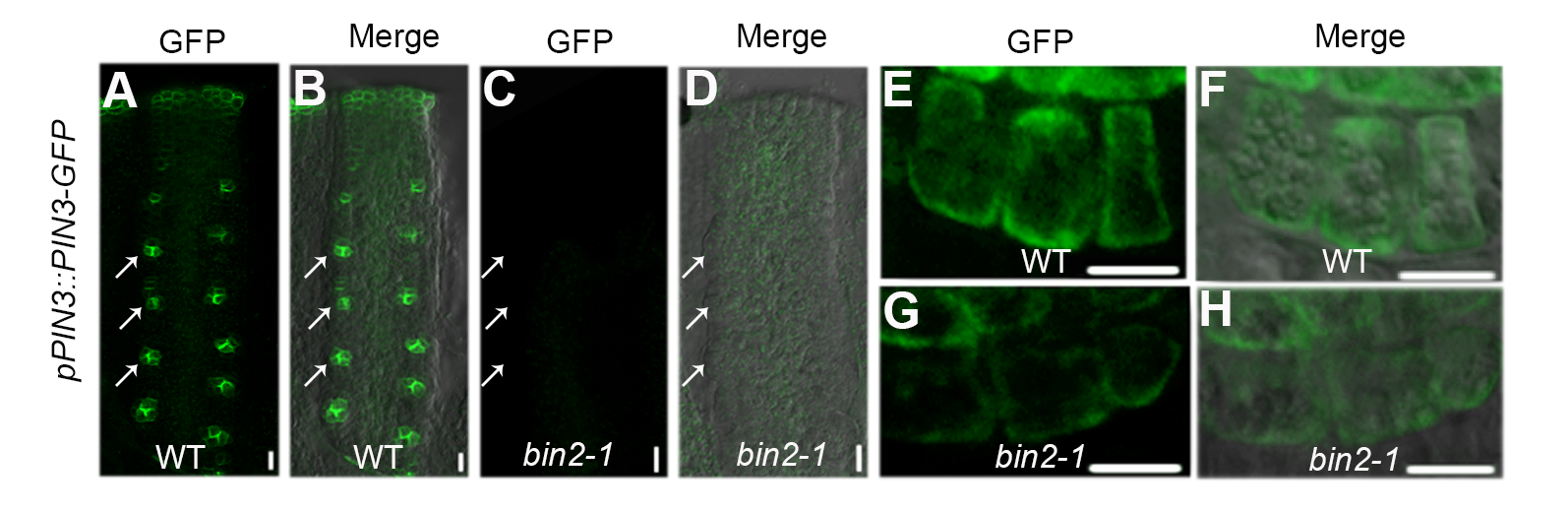

Supplement: S7 Fig — (A-D) pPIN3::PIN3-GFP expression in the ovule primordia of the wild-type (A-B), and bin2-1 mutant (C-D) at floral stage 9, respectively. White arrows represent the ovule primordia. (E-F) PIN3-GFP fused protein is uniformly localized in the columella cell boundaries of the wild-type root tip. (G-H) PIN3-GFP shows similar localization in the columella cell boundaries of the bin2-1 root tip to that of the wild-type. Bars: 10 μm. (TIF) [file pgen.1010077.s007.tif]
